# Towards Tsunami Early-Warning with Distributed Acoustic Sensing: Expected Seafloor Strains Induced by Tsunamis

**DOI:** 10.1007/s00024-026-03940-1

**Published:** 2026-03-19

**Authors:** Carlos Becerril, Anthony Sladen, Jean-Paul Ampuero, Javier Preciado-Garbayo, Miguel Gonzalez-Herraez, Fabian Kutschera, Alice-Agnes Gabriel, Frederic Bouchette

**Affiliations:** 1 Géoazur, Université Côte d’Azur, Observatoire de la Côte d’Azur, IRD, CNRS, Valbonne, France; 2 Polytechnic School, Department of Electronics, Universidad de Alcalá, Madrid, Spain; 3 Aragon Photonics Labs, Zaragoza, Spain; 4 Scripps Institution of Oceanography, University of California San Diego, La Jolla, CA USA; 5 Ludwig-Maximilians-Universität, Munich, Germany; 6 Geosciences-M/GLADYS, Université de Montpellier, CNRS, Montpellier, France

**Keywords:** Distributed acoustic sensing, tsunami, early warning, optic

## Abstract

Tsunami wave observations far from the coast remain challenging due to the logistics and cost of deploying and operating offshore instrumentation on a long-term basis with sufficient spatial coverage and density. Distributed Acoustic Sensing (DAS) on submarine fiber optic cables now enables real-time seafloor strain observations over distances exceeding 100 km at a relatively low cost. Here, we evaluate the potential contribution of DAS to tsunami warning by assessing theoretically the sensitivity required by a DAS instrument to record tsunami waves. Our analysis includes signals due to two effects induced by the hydrostatic pressure perturbations arising from tsunami waves: the Poisson’s effect of the submarine cable and the compliance effect of the seafloor. It also includes the effect of seafloor shear stresses and temperature transients induced by the horizontal fluid flow associated with tsunami waves. The analysis is supported by fully coupled 3-D physics-based simulations of earthquake dynamic rupture, seismo-acoustic waves, and tsunami (i.e., gravity wave) propagation. The strains from seismo-acoustic waves and static deformation near the earthquake source are orders of magnitude larger than the tsunami strain signal. We illustrate a data processing procedure to discern the tsunami signal. With enhanced low-frequency sensitivity on DAS interrogators (instrumental self-noise floor $\approx 1 \times 10^{-12}$), we find that, on seafloor cables located above or near the earthquake source area, tsunamis are expected to be directly observable with a sufficient signal-to-noise ratio within a few minutes of the earthquake onset. These results pave the way towards faster tsunami warning enabled by seafloor DAS.

## Introduction

Although several Tsunami Early Warning Systems (TEWS) are in operation worldwide, this is yet to be the norm, mainly due to the high cost associated with the installation and operation of offshore instrumentation with sufficient spatial coverage, density, and real-time data availability. To circumvent these challenges, and as the majority of recorded tsunamis worldwide are directly ascribed to earthquakes (Reid & Mooney, 2023), many TEWS rely on seismic data for source characterization. However, the energy released by the earthquake often is not a sufficient predictor of the tsunami intensity, which may lead to inaccurate early warnings and false alarms (Katsumata et al., 2021; Titov et al., 2016). Recent efforts to mitigate these issues include the integration of seismic observations with displacement data from land-based Global Navigation Satellite Systems (GNSS), to improve earthquake magnitude estimation and the inference of large-scale seafloor displacements, and thereby reduce false alarms in TEWS (Golriz et al., 2022). An effective system should use local wave measurements to characterize the tsunami and to allow the detection of tsunamis from other sources such as submarine landslides. For this reason, the basic sensor package for earthquake and tsunami early warning is a seismometer or accelerometer to detect ground shaking and a pressure gauge to detect tsunami waves (Wilcock et al., 2016).

To obtain reliable observations leading to faster tsunami confirmations, it is best to deploy instrumentation directly in the source region, including active subduction zones and areas prone to submarine landslides in volcanic systems. Such observation systems are crucial for protecting the population and improving our understanding of tsunami generation, which is less understood than the propagation process mainly due to scarce offshore and in-situ observations. The detection of tsunamis in the open ocean, achieved by monitoring variations in water pressure, is significantly challenged by the relatively modest amplitudes of tsunamis typically around a meter, even for the largest events - and by the logistical complexities and expenses involved in maintaining instruments in remote oceanic locations on a long-term/permanent basis. Additionally, ensuring dense and extensive spatial coverage across all potential tsunami sources complicates this endeavor, making it difficult to rapidly and robustly estimate a tsunami’s potential impact. Furthermore, little is known about spatial variations of tsunamis, because too few dense array measurements of tsunamis are available so far (Kohler et al., 2020). As the population grows in coastal regions, the recurrence of tsunami tragedies underscores the urgent need for better detection methods and early warning systems.

Direct tsunami observations are made mostly by coastal tide gauges and fixed moorings or buoys located offshore, such as the Deep-ocean Assessment and Reporting of Tsunamis (DART) system. The current network of 77 DART stations worldwide (as of March 2025) has a sparse sensor density and a limited spatial footprint. Each DART station consists of a transmitter surface buoy and an anchored seafloor pressure sensor (National Oceanic and Atmospheric Administration, 2023). With an approximate cost of US$ 0.5M per station and high maintenance and repair costs, compounded by the remote location of the buoys and required ship operations, densifying DART instrumentation requires a financial commitment which can be prohibitive for developing countries (Bernard & Titov, 2015). Moreover, the DART system is primarily designed for providing forecasts in the case of transoceanic or far-field tsunamis, but not for regional and local tsunamis. A confirmation of tsunami and an evaluation of its amplitude are obtained once the recordings from the closest station are analyzed, which may often take two to three hours (Mungov et al., 2013). Tide gauges are typically located inside harbors and bays, and hence can only have a limited contribution to early warning. Furthermore, the tsunami signal of tide gauge records are often filtered or distorted by the shallow coastal water depth, which makes it difficult to extract detailed information about a tsunami (Saito, 2019).

In rare cases, direct tsunami observations are made by cabled observatories: the North-East Pacific Time-series Undersea Networked Experiments system (NEPTUNE) in Canada (Heidarzadeh, 2019), the Dense Oceanfloor Network System for Earthquakes and Tsunamis (DONET), the N- and S-NET systems in Japan. DONET consists of 51 stations over a length of 800 km, whilst S-NET is the world’s largest seafloor observation network, consisting of 150 cable-linked seismic and tsunami sensors covering an area of 1000 km x 300 km. Both are deployed on the slopes of subduction zone trenches and in rupture-prone regions, such as those associated with the historical Tonankai and Nankai earthquakes (Aoi et al., 2020). These observation systems have been used to detect several tsunamis of various sizes based on changes in water pressure (Titov et al., 2005; Tsushima et al., 2012), including the disastrous tsunami of the 2011 Tohoku-oki earthquake (Maeda et al., 2011; Saito et al., 2011). Although capable of carrying robust, long-term observations, cabled observatories require a substantial financial investment, especially to achieve a wide and complete spatial coverage on the scale of subduction zones.

Distributed Acoustic Sensing (DAS) is a technology that re-purposes existing fiber optics in telecommunication cables into long and dense arrays of longitudinal strain sensors (Zhan, 2020). DAS can record external inputs that deform fiber optic cables in a broad frequency range (Paitz et al., 2020), with a current maximum distance range of around 150 km (Waagaard et al., 2021). These capabilities have motivated a decade of applications in subsurface imaging and microseismicity monitoring for energy production and carbon sequestration. DAS arrays have recorded microearthquakes, regional earthquakes, teleseisms, and infrastructure signals. Analysis of these wavefields is enabling earthquake seismology where traditional sensors were sparse, as well as structural and near-surface seismology. These studies have improved our understanding of DAS instrument response through comparison with traditional seismometers. More recently, DAS has been used to study cryosphere systems, marine geophysics, geodesy, and volcanology (Lindsey & Martin, 2021).

Several key requirements for TEWS are inherent attributes of DAS: low data latency, high spatial density, and relatively low cost, often dominated by the DAS interrogator equipment cost ($$\sim$$ US$ 150k). Currently commercially available DAS interrogators sense strain with meter-scale spatial resolution over cable spans of up to 150 km and deliver data on land in real-time (Fernandez-Ruiz et al., 2022; Sladen et al., 2019; Williams et al., 2023). Coupling these attributes with the existing (and still expanding) global coverage of the transoceanic telecommunication cable network (Brenne et al., 2024), positions DAS as a sensor package to consider for TEWS. It provides a cost-effective means of deploying instrumentation to monitor offshore locations such as subduction zones, and can also provide several measurements per tsunami wavelength to better study the characteristics of tsunamis.

The earliest reports on seafloor DAS for Earth science documented signals induced by the swell (Lindsey et al., 2019; Sladen et al., 2019; Williams et al., 2019). In particular, (Sladen et al., 2019) showed that the depth-dependence of the signal amplitude is consistent with the pressure depth-dependence from the linear theory of surface gravity waves. This demonstrated that DAS can record surface gravity waves, although at much shorter periods than tsunami waves.

So far there are two reported detections of a tsunami with seafloor DAS. Xiao et al. (2024) made use of 60 km of cable off the northwestern US coast to detect a far-field tsunami, which produced a small tsunami wave height of $\sim$ 6 mm at a DART station, and reached $\sim$ 1 cm at one end of the monitored fiber. The signal-to-noise ratio of the detected tsunami phase was low, even after beamforming. Tonegawa (2024) detected high-frequency tsunami signals in the 5–30 mHz frequency range on a 128 km long submarine cable located approximately 300 km west of a tsunamigenic seismic source. The authors allude to the challenge of distinguishing tsunami signals from background infragravity waves, as well as the uncertainties in attributing strain to specific contributions from pressure-induced and deformation-induced components, and temperature effects. Both reports underscore the feasibility of DAS to detect tsunami waves, whilst referencing key aspects of DAS that need to be addressed to ascertain tsunami wave detection with sufficient signal-to-noise ratio to be integrated into TEWS.

DAS is most sensitive to strain along the cable axis. Given that cables are typically laid or buried along the seafloor, they can primarily perform as horizontal arrays of sensors recording horizontal strain, which is not a quantity that has been considered before in tsunami studies. The question of sensitivity at low frequencies also merits attention given that DAS instrumentation usually has lower performance at long periods typical of a tsunami (>100 s) (Fernández-Ruiz et al., 2019).

The goal of this work is to develop a theoretical understanding of the strain amplitudes that can be induced on seafloor cables by tsunami processes, to quantify the potential contribution of DAS to TEWS. Our analysis anticipates that two effects induced by the hydrostatic pressure perturbation of tsunami waves, namely the elastic deformation of the seafloor and the Poisson effect on the cable, are the primary mechanisms through which DAS systems may record the passage of tsunami waves. The sub-horizontal deep water flow induced by tsunami waves can also generate DAS signals, but we find their potential contribution to be minor in deep waters.

The remainder of the paper is organized as follows. In Sect. 2, we review recently reported developments in the range and sensitivity of DAS at low frequencies, intended to enable improved accuracy over long distances in the mHz frequency range of interest in tsunami studies. Then, we present a theoretical analysis of multiple mechanisms by which seafloor cable strains can be induced by tsunami waves: the seafloor elastic compliance and Poisson’s effects arising from the hydrostatic pressure changes (Sect. 3), and shear strain at the seafloor induced by the lateral water flow associated with tsunami waves (Sect. 4). With the aforementioned hydrostatic and shear sources of strain, we then simulate the expected strain signal amplitude at different depths for a propagating tsunami wave (Sect. 5). In Sect. 6, we consider the possible influence of temperature fluctuations at the seafloor induced by the lateral water flow associated with tsunami waves. Finally, in Sect. 7, we carry a 3-D fully coupled simulation of earthquake dynamic rupture including seismic, acoustic, and gravity wave (tsunami) propagation; this simulation allows us to evaluate the capability of a DAS-driven TEWS system even in the near-field of a large earthquake ruptures.

## Sensitivity of Advanced DAS Instrumentation at Tsunami Frequencies

DAS interrogators inject a laser pulse into an optical fiber and exploit the backscattered light due to Rayleigh scattering by inherent refractive index heterogeneities along the fiber. Techniques such as Optical Time Domain Reflectometry (OTDR) determine the location of such heterogeneities along the fiber. Deformation and temperature perturbations of the fiber due to environmental changes cause phase changes in the backscattered light, which can be detected via the phase-sensitive version of OTDR ($\Phi$OTDR). Optical interferometry is used to quantify the phase differences between the backscattered light produced by two pulses injected at different times. These raw measurements of optical phase shifts are integrated along discrete cable segments, the “gauge length”, and then converted to position-resolved strain or strain-rate at each “DAS channel”.

In $\Phi$OTDR, the dynamic range is limited because phase differences lie between $-\pi$ and $\pi$ (Diaz-Meza et al., 2023; Masoudi et al., 2013). Furthermore, this approach requires optical phase-sensitive detection methods, which can be complex and add stringent performance requirements on the optical hardware. An advantageous approach is to employ linearly chirped pulses, known as Chirped-pulse DAS, where the instantaneous frequency of the pulse varies linearly with time across the pulse duration, thereby performing frequency-based interrogation in a single pulse. This chirped-pulse approach of phase-sensitive optical time-domain reflectometry (CP-$\Phi$OTDR) allows a frequency-to-time mapping of the backscattered light, which enables detection of phase shifts by determining the temporal shift between reference and measurement acquisitions via direct amplitude photodetection, without the need to resort to phase-sensitive photodetection techniques or phase unwrapping (Pastor-Graells et al., 2016, 2017). In contrast to $\Phi$OTDR, the measurable dynamic range of CP-$\Phi$OTDR is a function of the spectral characteristics of the chirped pulse (Fernández-Ruiz et al., 2019): the maximum strain variation between pulse and reference, $\Delta \epsilon _{max}$, is limited by the largest temporal shift that can be measured accurately (Bhatta et al., 2019) given the frequency content of the chirped pulse.

**Figure 1:**
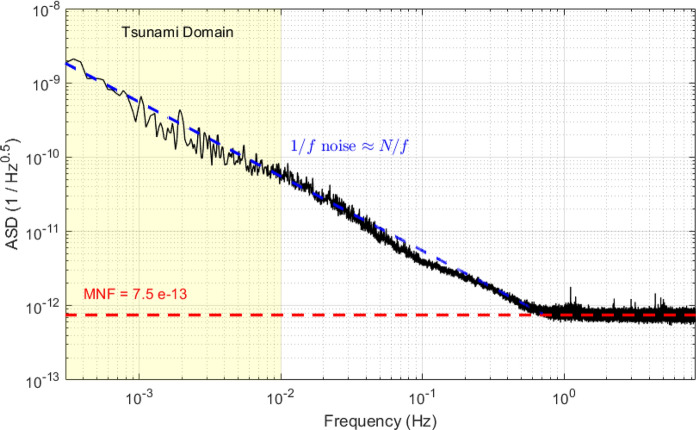
Mean Amplitude Spectral Density (ASD) calculated from a 24,000 s. time series of the noise recorded by a chirped-pulse DAS (CP-$\Phi$OTDR) in a laboratory test on a static fiber under limited environmental noise. The frequency of acquisition was 2 kHz and decimated to 100 Hz for subsequent processing, with a 50 m gauge length and a 10 m spatial sampling interval. The black curve represents the mean of individual ASD curves calculated over 200 consecutive channels, spaced 10 m apart along the fiber. The yellow-shaded region represents the range of frequencies associated with tsunami waves. Below 1 Hz, the ASD has an approximate inverse frequency dependence $\sim N/f$ (blue dashed line), interpreted as due to environmental noise. At higher frequencies, the ASD is approximately frequency-independent (horizontal red dashed line), with a median noise floor (MNF) considered as the instrumental noise floor, not affected by environmental noise

Figure 1 shows the results of a laboratory test in which a CP-$\Phi$OTDR was used to interrogate a 10 km-long single-mode fiber, isolated acoustically and thermally. The interrogator is a pre-commercial version manufactured by Aragon Photonics with enhancements in the long-term stability of the electro-optical components. A linear frequency sweep was applied along narrow optical pulses. The backscattered signal was sampled by a high-bandwidth digitizer. Then, linear signal processing was performed to compute the strain for each DAS channel. Throughout the experiment, the temperature inside the fiber coil was continuously monitored and remained stable, with variations kept below $0.1^{\circ }$C-the resolution limit of the digital thermometer used.

The Amplitude Spectral Density (ASD) of the recorded data (Fig. 1) shows two main features: an approximately inverse frequency decay (ASD $\approx N/f$) at frequencies lower than 1 Hz, and a frequency-independent level at higher frequencies. These two regimes are interpreted as follows.

The 1/*f* regime at low frequencies results from a combination of environmental noise and instrumental noise induced by reference updates triggered by environmental drifts. Relying on a reference measurement to evaluate the phase change of the backscattered light has the drawback that any large strain signal, accumulated temperature drift, or laser frequency noise can cause large deviations in the phase shift. When these phase deviations reach the dynamic range, the current reference trace becomes potentially invalid and the instrument resorts to updating the reference. The reference updates unavoidably introduce error accumulation over time (noise is integrated with each cumulative variation measurement) (Magalhães et al., 2022), which produces a 1/*f* noise component. With the configuration for the chirped-pulse DAS used to obtain Fig. 1 (1 GHz chirp content and 2 kHz acquisition rate), the dynamic range is $\Delta \epsilon _{max} \sim \pm 0.4\times 10^{-6}$. In the laboratory experiment reported in Fig. 1, the 1/*f* noise is most probably the result of environmentally induced drift due to limitations in the environment control used to mitigate acoustic and temperature fluctuations on the measured fiber.

The high-frequency plateau in Fig. 1 corresponds to the noise floor limit due only to instrumental sources. The instrumental noise floor limit for CP-$\Phi$OTDR DAS is determined by the Cramer-Rao lower bound $\sigma _{CRLB}$ of performance for the optical configuration used (i.e. chirp bandwidth, signal-to-noise ratio of the optical trace and pulse duration) (Costa et al., 2019) and by the frequency of acquisition $f_{a}$ (Fernández-Ruiz et al., 2019) as:

1 $$\begin{aligned} ASD = \frac{\sigma _{CRLB}}{\sqrt{f_{a}/2}} \end{aligned}$$

With $f_{a} =2$ kHz used in Fig. 1, $ASD \approx 7.5 \times 10^{-13}$ $1/\sqrt{Hz}$. In particular, Eq. 1 shows how the instrumental noise floor can be decreased by increasing the acquisition frequency.

These results of laboratory experiments can be transposed to a seafloor DAS acquisition situation for tsunami monitoring as follows. The acquisition frequency is limited by the round-trip time of the optical pulse along the length of fiber being monitored. With an acquisition rate of 2 kHz it is possible to monitor a fiber length up to 50 km. For tsunami monitoring, we would typically consider interrogating a 100 km length of fiber, to span a substantial distance between the coast and the subduction trench, which requires a sampling rate up to 1 kHz. Then, based on equation 1 and the noise floor observed in Fig. 1, the (high-frequency) instrumental noise floor would be $ASD\approx 1.06 \times 10^{-12}$ $1/\sqrt{Hz}$. At low frequencies, the amplitude of the 1/*f* noise would be controlled by the environmental background temperature and strain fluctuations intrinsic to ocean-bottom environments. In deep ocean bottom, temperature fluctuations are generally far smaller than in the laboratory environment of Fig. 1, and we expect the instrument noise floor limit obtained at high frequencies to extend to frequencies lower than 1 Hz. DAS based observations in different ocean basins (Ide et al., 2021; Pelaez Quiñones et al., 2023; Williams et al., 2023) could mainly detect temperature perturbations in deep water when internal waves would interact with the slope (i.e, not apparent on flat cable sections) and at periods $> 1000$ s; this will be discussed in more detail in Sect. 6. Even if the cable to be monitored does not have flat sections and is in shallow depths ($\sim 100$ m), the typical speed of internal wave perturbations is at least 2 orders of magnitude slower (0.1 m/s) than tsunami waves ($\sim 30$ m/s) making it possible to filter these perturbations out, as illustrated in Sect. 7. Thus, hereafter we adopt $1.06 \times 10^{-12}$ $1/\sqrt{Hz}$ as reference DAS sensitivity at all relevant frequencies. We separately consider the effect of seafloor background temperature fluctuations in Sect. 6.1.

## Strain of Seafloor Optical Fibers from Water Pressure Loading

**Figure 2:**
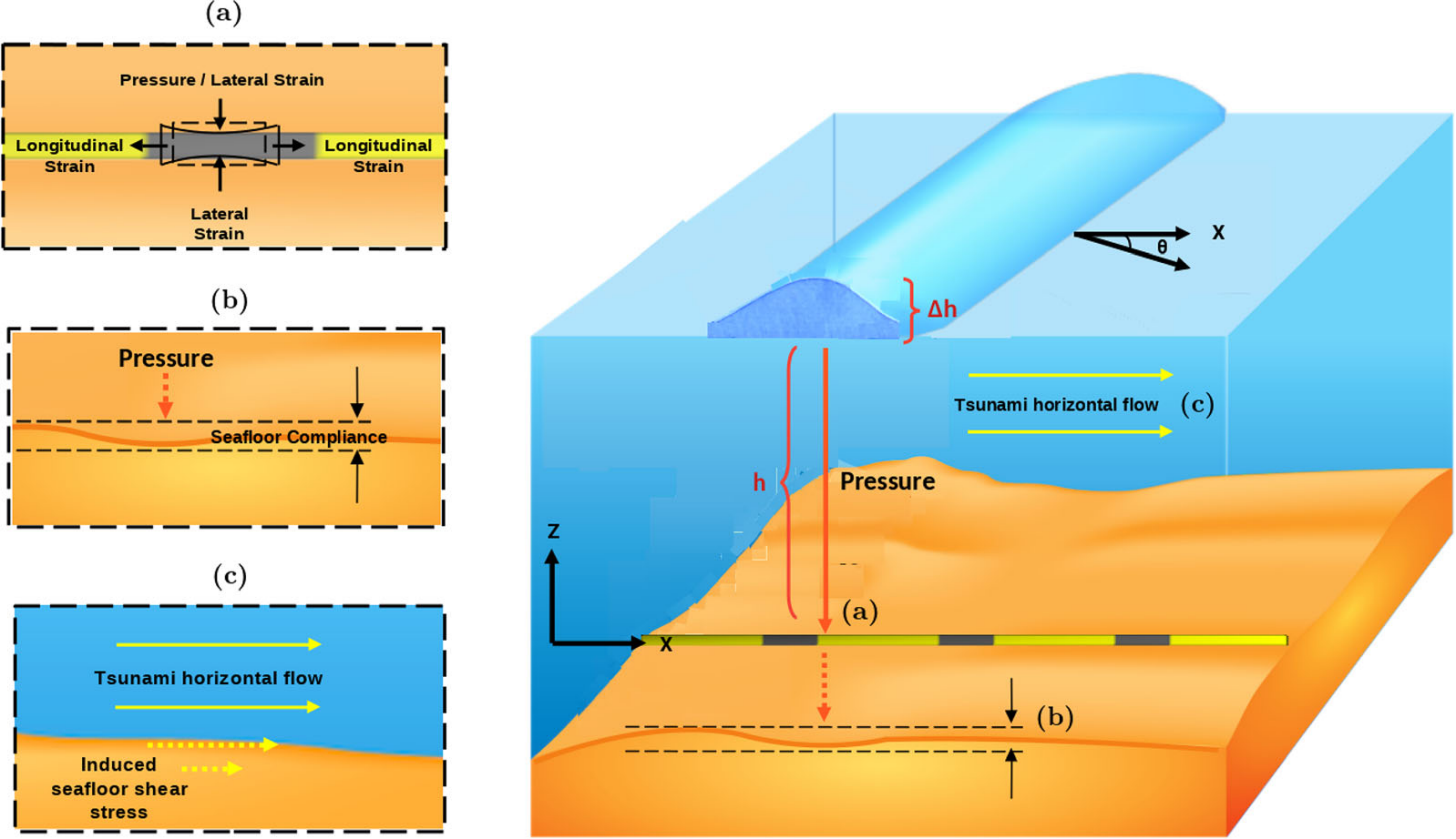
Illustration of a submarine cable in the presence of a tsunami wave propagating at an angle $\theta$ to the *X*-axis, which is parallel to the seafloor cable. Vertical pressure loading due to changes in the water column, $\Delta$h give rise to **a** longitudinal elongation of the cable due to the Poisson effect and **b** deformation of the seafloor (compliance). The horizontal flow motion beneath the tsunami wave induces **c** shear stress on the seafloor surface. Also shown is the coordinate system convention and definition of relevant variables

Here, we estimate the expected amplitude of seafloor horizontal strains that could be generated by the hydrostatic pressure variations induced by the changes in water height via two effects: the compliance effect of the seafloor and the Poisson’s effect of the cable; both illustrated in Fig. 2.

### Compliance Effect

The water pressure perturbation induced by a tsunami wave acts on the seafloor and deforms it elastically (Fig. 2b). The problem of determining the strain of an elastic half-space caused by a normal force on its surface was first considered by J. V. Boussinesq. In this formulation, the solid Earth is approximated by a homogeneous, isotropic, linear elastic half-space. Neglecting the spherical geometry of Earth is adequate because we consider tsunami wavelengths that are short compared to Earth’s radius. Furthermore, as the tsunami velocity is far slower than the acoustic velocity, the inertia term of the moving medium is neglected. Details of the solution are given by (Steketee, 1958), from which we take the expression for horizontal displacement parallel to the cable axis ($\bar{\textit{u}}_{x}$) in the wavenumber domain (spatial Fourier transform):

2 $$\begin{aligned} \bar{\textit{u}}_{x} = \textit{i} k_x \frac{\bar{P}}{2\mu k^2}\left( 1-\frac{1}{\alpha }+k z\right) e^{-k z} \end{aligned}$$

where $\bar{P}$ is the normal pressure with an arbitrary spatial distribution, *z* is the vertical coordinate, defined as pointing upwards,

3 $$\begin{aligned} \alpha =\frac{\lambda +\mu }{\lambda +2\mu }, \end{aligned}$$

where $\lambda$ and $\mu$ are Lamé’s elastic moduli, and $k=\sqrt{k_x^2 + k_y^2}$ is the amplitude of the wavenumber vector $(k_x,k_y)$ along the horizontal plane, where we focus on plane tsunami waves with finite non-zero wavenumber *k*. The horizontal displacement at the seafloor ($z=0$) is

4 $$\begin{aligned} \bar{u}_{x}= \textit{i}k_x\frac{\bar{P}}{2\mu k^2}\left( 1-\frac{1}{\alpha }\right)= -i k_x \frac{\bar{P}}{2 k^2(\lambda +\mu )} \end{aligned}$$

The strain along the cable axis (*x*-axis), which is the quantity measured by DAS, is $\epsilon _x=\frac{du_x}{dx}$. Since its Fourier transform is $\bar{\epsilon }_x=i k_x\bar{u}_x$, we obtain:

5 $$\begin{aligned} \bar{\epsilon }_x = \frac{k_x^2}{k^2} \frac{\bar{P}}{2 (\lambda +\mu )} \end{aligned}$$

Defining $\theta$ as the angle between the wavenumber vector (the tsunami wave propagation direction) and the cable axis, we have $k_x/k = \cos \theta$ and $\bar{\epsilon }_{x}=\frac{\bar{P}\cos ^2\theta }{2(\lambda +\mu )}$. Taking the inverse Fourier transform of $\bar{\epsilon }_{x}$, the amplitude of seafloor strain oscillation induced by a single tsunami plane wave is

6 $$\begin{aligned} \epsilon _{x}=\frac{P\cos ^2\theta }{2(\lambda +\mu )} \end{aligned}$$

The $\cos ^2$ term is a well-known angular sensitivity feature of DAS (Mateeva et al., 2014). For the case of a wave propagating perpendicular to the fiber, although the pressure is time-varying, the pressure along the cable is uniform (the gradient along the fiber is zero), thus not inducing longitudinal strain. The case of uniform pressure over the seafloor is not relevant for our work. We obtain a generic estimate of the amplitude of this effect by taking the average over all possible tsunami wave directions:

7 $$\begin{aligned} \epsilon _{x\_avg} = \frac{P}{4(\lambda +\mu )} \end{aligned}$$

### Poisson’s Effect

Another potential contribution to the strain signal detected by DAS is from Poisson’s effect: water pressure perturbations cause radial compression of the submarine cable, which in turn induces longitudinal elongation of the cable through Poisson’s effect (Fig. 2a). We assume that water pressure acts isotropically on the whole circumference of the cable, neglecting the fact that the cable might be partially buried. Representing the cable as an effective homogeneous medium with effective Young’s modulus *E* and Poisson’s ratio $\nu$, its longitudinal strain due to Poisson’s effect is given by (Tatekura et al., 1982):

8 $$\begin{aligned} \epsilon _x = 2\frac{1-2\nu }{E} P \end{aligned}$$

The cable’s sensitivity to pressure is highly dependent on the cable design. For an optical submarine cable assembly, $E \sim 5 - 50$ GPa and $\nu \sim 0.2 - 0.25$ (Kojima et al., 1982; Tatekura et al., 1982). The lower values of *E* and larger values of $\nu$ correspond to less armored cables. For a submarine cable with mid-range elastic properties ($\nu = 0.23$ and $E = 25$ GPa) the Poisson’s effect sensitivity is $\epsilon _x \sim 4.3 \times 10^{-11}\ \Delta P$, which falls within expectations based on previously reported theoretical estimates (Budiansky et al., 1979; Haavik, 2022).

### Expected Strain Signal from Hydrostatic Pressure

Here we combine the two pressure effects and estimate their total expected contribution to seafloor DAS strain as a function of tsunami wave height and frequency. We stay within the linear regime of the tsunami theory. This framework assumes that the wave amplitude (typically less than 10 m) is much smaller than the wavelength. This assumption is generally violated only during the final stage of wave breaking near the coast, or under extreme tsunami generating conditions. We neglect the feedback of seafloor compliance on tsunami waves, which is significant at much longer periods and propagation distances than considered here (Tsai et al., 2013); we thus adopt the conventional tsunami theory with a rigid seafloor. We assume an incompressible, homogeneous, and non-viscous ocean, subject to a constant gravitational field, with uniform water depth *h*.

Within this framework, the frequency $\omega$ and wavenumber *k* of a sea surface gravity wave satisfy the dispersion relation

9 $$\begin{aligned} \omega ^2 = g k\tanh (kh) \end{aligned}$$

where *g* is the gravitational acceleration. For a given frequency within the range of interest in tsunami studies, and for sea depths *h* ranging from 100 m to 6 km, we solve the above relation for the corresponding *kh*. The amplitude of the pressure variation $\Delta P$, induced by a sea surface height anomaly (SSHA) of amplitude $\Delta h$, at a vertical position *z* relative to the seafloor, is

10 $$\begin{aligned} \Delta P(z) =\rho g \Delta h\frac{\cosh (kz)}{\cosh (kh)} \end{aligned}$$

Where $\rho$ is the water density. The seafloor pressure amplitude (at $z=0$) is thus

11 $$\begin{aligned} \Delta P =\frac{\rho g\Delta h}{\cosh (kh)} \end{aligned}$$

The $\cosh$ term induces a low-pass filter effect: only long wavelengths generate significant seafloor pressure changes. As a validation of this linear theory of surface gravity waves with DAS data, (Sladen et al., 2019) showed that the depth-dependence in Eq. 11 is consistent with the decay with depth of the amplitude of DAS signals from swell. An estimate of tsunami amplitude ($\Delta h$) via DAS observations ($\epsilon$) due to seafloor pressure variations can be attained by combining Eqs. 6, 8 and 11:

12 $$\begin{aligned} \Delta h = \frac{ \epsilon _x \cosh (kh) }{ \rho g \left( \dfrac{ \cos ^2 \theta }{ 2(\lambda + \mu ) } + \dfrac{ 2(1 - 2\nu ) }{ E } \right) } \end{aligned}$$

The terms in the denominator come from independent material properties of the cable assembly (Young’s modulus and Poisson’s ratio) and the elastic properties of the seafloor (Lamé’s parameters), which can be further calibrated with field data. Such field calibration can also narrow the angular range $\theta$ may occupy based on the direction where the tsunami energy is likely to be more prevalent for a given bathymetry, or as will be exemplified in Sect. 7 for the case of an earthquake-tsunami, the fault geometry and orientation.

We calculate seafloor pressure for values of $\Delta h$ (SSHA) of 0.1 and 1 m representative of intermediate and large-size tsunamis in the open ocean. We use the resulting pressure to calculate the longitudinal cable strain due to the compliance effect and Poisson’s effect through Eqs. (7) and (8), respectively. We add the two effects to obtain the total DAS strain due to pressure perturbations. To evaluate the compliance effect, for a given frequency, we consider elastic parameters $\lambda$ and $\mu$ from the Preliminary Reference Earth Model (PREM) (Dziewonski & Anderson, 1981) averaged down to a depth proportional to the tsunami wavelength, namely 1/*k* (Crawford, 2004). For wavelengths between 10 and 100 km, Eq. 6 yields a sensitivity to pressure via the compliance effect of $\epsilon _x \sim (5.5 \times 10^{-11} - 4.8 \times 10^{-12})\ \Delta P$.

With the elasticity parameters considered in this study for the cable assembly and those from the PREM model, the sensitivity to Poisson’s (estimated in the previous section) and compliance effects are very similar. One feature that might help distinguish their relative contributions in field data is that only the compliance effect depends on the wave propagation direction ($\theta$ is involved in Equation (6) but not in Eq. (8)). Another distinguishing factor pertains to the burial of the cable. For typical magnitudes of low frequency ground motions, the mechanical coupling is stronger between a buried optical fiber and the seafloor (Reinsch & Thurley, 2017), whilst the inverse is true for the susceptibility of a buried cable to Poisson’s effect, as this depends on the transfer of fluid pressure to the optical fiber (Becker et al., 2017). Such coupling coefficient should be determined based on the installation conditions of the cable, and further calibrated using field data (e.g. tides).

The sum of Poisson’s and compliance effects yields an order-of-magnitude theoretical estimate of pressure sensitivity of $\epsilon _x/\Delta P \sim 10^{-10}$ $\hbox {Pa}^{-1}$. This is similar to or somewhat smaller than recent empirical estimates: $\epsilon _x/\Delta P \sim 1\times 10^{-10}$ $\hbox {Pa}^{-1}$ in (Meulé et al., 2024), $\epsilon _x/\Delta P \sim 5\times 10^{-10}$ $\hbox {Pa}^{-1}$ in (Williams et al., 2023), and $\epsilon _x/\Delta P \sim 10^{-9}$ $\hbox {Pa}^{-1}$ in (Glover & Wengrove, 2024). The latter estimate was obtained using a bespoke cable in shallow water ($\le$15 m) and for relatively short wavelengths associated to frequencies in the 0.04−0.3 Hz band, thus the difference can be partly attributed to enhanced coupling, the lower stiffness of shallow sediments and to larger contributions from shoaling and seafloor shear effects. An additional order-of-magnitude verification is based on DAS signal amplitudes reported by Sladen et al. (2019) for surface gravity waves recorded at shallow depths (< 100 m). Considering their observed strain rates (extrapolated) at zero depth $\dot{\epsilon } \sim 2\times 10^{-7}$ $\hbox {s}^{-1}$, their dominant period $\sim 10$ s, and $\epsilon _x/\Delta P \sim 10^{-10}$ $\hbox {Pa}^{-1}$, yields $\Delta P \sim 10^4$ Pa. This pressure corresponds to $\Delta h \sim 1$ m, which agrees in order-of-magnitude with reported wave heights in the same region (Guerin et al., 2022).

**Figure 3:**
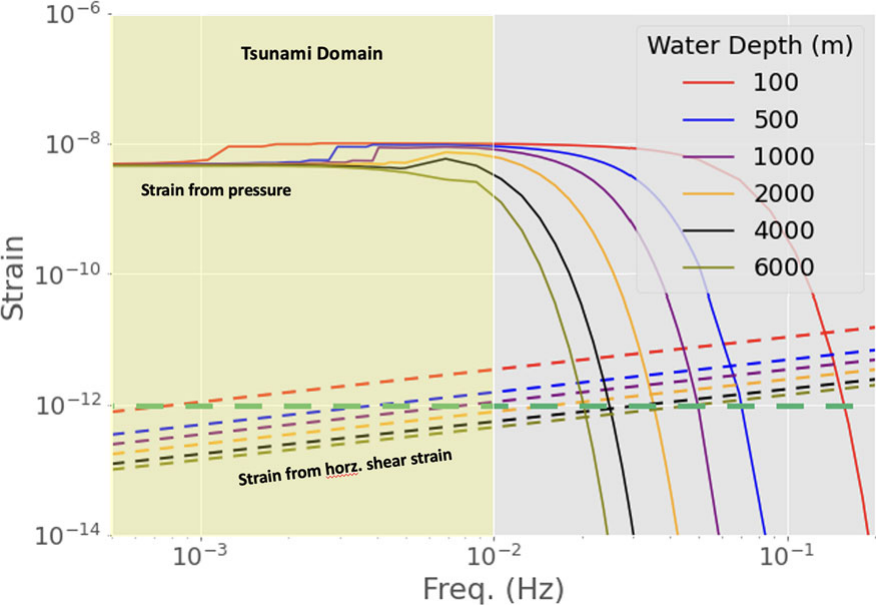
Expected strain amplitudes for a tsunami height (sea surface height anomaly, SSHA) of 0.01 m at various water depths *h* as indicated on the color legend. Solid lines correspond to the strain from hydrostatic pressure at the seafloor (contributions from Poisson effect on the cable and seafloor compliance). Dashed lines correspond to the strain due to the shear stress from the horizontal flow motion beneath a tsunami wave. The green dashed line represents the noise floor amplitude for the enhanced CP-DAS unit described in chapter 2. The yellow-shaded region encompasses the range of frequencies corresponding to tsunami waves. The attenuation of the higher frequency components ($>10^{-2}$ Hz) with depth illustrates the evanescent nature of the high-frequency pressure field with depth, and the progressive transition from shallow water to deep water approximation. For the cable assembly, nominal values of *E*=25 GPa and $\nu$=0.23 were used for Young’s modulus and the Poisson’s ratio, respectively. The seafloor is modeled as a stratified solid with elastic properties based on the PREM model. The ocean has a density $\rho$ = 1030 kg/m^3^, the ocean has an acoustic wave speed of $c_p$ = 1500 m/s and the water particle viscosity $\nu _s$ = 1E-6 m^2^/s. Simulation results for 1 m SSHA can be inferred from this figure as the corresponding pressure (and strain) scales linearly

The solid lines in Fig. 3 show the resulting strains as a function of frequency, along with the median noise floor illustrated in Fig. 1 for the calibrated version of a chirped-pulse DAS instrument. The estimated strain amplitudes yield signal-to-noise ratios (SNR) larger than 10 for a wave height of 0.01 m, suggesting that the calibrated CP-DAS could reliably detect signals from the pressure induced by tsunami waves. The stacking of multiple DAS channels could further improve detectability; with typical DAS gauge lengths $\sim 10$ m, it would be feasible to stack 100 channels along cable segments of $\sim 1$ km length that are still much shorter than a typical tsunami wavelength, potentially improving SNR by a factor of 10.

## Seafloor Strain from Shear Stress Beneath a Tsunami Wave

### Sea Bottom Shear Stress Induced by a Tsunami Wave

The horizontal displacement of water masses by a tsunami wave produces seafloor shear stresses, which in turn can induce longitudinal strain on a seafloor cable (Fig. 2c). The horizontal and vertical particle velocities of a tsunami wave of frequency $\omega$ in the linear wave theory are (Dean & Dalrymple, 1991):

13 $$\begin{aligned} \begin{aligned} \textit{v}_x (x,t)&= A\frac{\cosh (kz)}{\sinh (kh)}\cos {(kx- \omega t)}\\ \\ \textit{v}_z (x,t)&= A\frac{\sinh (kz)}{\sinh (kh)}\sin {(kx-\omega t)} \end{aligned} \end{aligned}$$

where *z* is vertical position relative to the seafloor, and

14 $$\begin{aligned} \textit{A} = \frac{\Delta h}{2}\omega \end{aligned}$$

The vertical velocity is maximal at the ocean surface and decays exponentially to zero at the seafloor. In contrast, the horizontal velocity remains relatively constant through the water column. Its amplitude at the seafloor ($z=0$), assuming shallow-water conditions, is

15 $$\begin{aligned} \textit{v}_x (x) = \frac{\Delta h\ \omega }{2\sinh (kh)} \approx \frac{\Delta h\ \omega }{2kh} = \frac{\Delta h}{2}\sqrt{\frac{g}{h}} \end{aligned}$$

The resulting bottom shear stress is

16 $$\begin{aligned} \tau _w = \frac{\rho }{2} \textit{f} \textit{v}_x^2 \end{aligned}$$

where *f* is the friction coefficient. Under a tsunami wave, *f* is determined by the flow regime on the bottom boundary layer. According to the analysis by (Tinh & Tanaka, 2019), for most of the propagation phase, the tsunami-induced bottom boundary layer shows an unsteady behavior and resembles that induced by wind-driven waves, even under long-period wave motion; with the transition from wave to steady-motion typically occurring only a few meters from shore. The bottom boundary layer at the tsunami source is within the laminar regime and subsequently, a transition occurs to smooth turbulence during the shoaling process, with a transition from smooth to rough turbulent region at shallow depths. In this study, the full-range equation proposed by ( Tanaka et al. (2020), Eq.18) for the wave–current combined motion, is implemented to compute the wave friction coefficient under shoaling tsunami, $\textit{f}_w$, given that it yields a smoothly interpolated value for each of the aforementioned flow regimes including the transitional domain. The full-range equation is a function of the wave-induced velocity $\textit{v}_x$, the angular frequency of the wave, $\omega$, the roughness of the seabed material (sand grain diameter), and the viscosity of the fluid. The horizontal strain imparted onto both the seafloor and the cable by the applied shear stress (equation 16) is determined next.

### Shear-Induced Strain on the Seafloor

The seafloor shear stress $\tau _w$ can deform elastically the seafloor, resulting in a horizontal seafloor strain $\epsilon _{xx}$. To estimate this effect theoretically we begin with the Fourier domain expression found in (Barbot & Fialko, 2010) for the displacement field of a semi-infinite elastic solid induced by an arbitrary distribution of surface tractions, which also accounts for a buoyancy restoring force at the surface. It consolidates the solution for the Boussinesq’s and Cerruti’s problems, which deal with normal and tangential (shear) loads, respectively. From their equation 29, considering only shear loads (setting the normal load to zero) and ignoring the gravity effect (setting their $\Gamma$ coefficient to zero), the horizontal displacement at the seafloor ($z=0$) in the direction of the cable axis (*x*) is

17 $$\begin{aligned} \bar{u}_x = -2B_x k^2 + \alpha k_x (B_x k_x + B_y k_y) + \alpha i k_x k B_z (1 - \alpha ^{-1}) \end{aligned}$$

where the constants $B_i$ depend on the applied shear loads as

18 $$\begin{aligned} B_x&= -\frac{\bar{\tau }_x}{2 \mu k^3}, \quad B_y = -\frac{\bar{\tau }_y}{2 \mu k^3}, \quad B_z = \frac{i (1 - \alpha )(\bar{\tau }_x k_x + \bar{\tau }_y k_y)}{2 \mu \alpha k^4}. \end{aligned}$$

Thus,

19 $$\begin{aligned} \bar{u}_x = \frac{\bar{\tau }_x}{\mu k} - \frac{\alpha k_x (\bar{\tau }_x k_x + \bar{\tau }_y k_y)}{2 \mu k^3} - \frac{k_x (1 - \alpha ) (\bar{\tau }_x k_x + \bar{\tau }_y k_y)}{2 \mu k^3} (1 - \alpha ^{-1} ) \end{aligned}$$

After simplifications:

20 $$\begin{aligned} \bar{u}_x = \frac{\bar{\tau }_x}{\mu k} - \frac{k_x (\bar{\tau }_x k_x + \bar{\tau }_y k_y)}{2 \mu k^3} (2 - \alpha ^{-1}) \end{aligned}$$

Considering the angle $\theta$ between the flow direction and the cable axis, we write $\bar{\tau }_x=\bar{\tau }\cos {\theta }$ and $\bar{\tau }_y=\bar{\tau }\sin {\theta }$, where $\bar{\tau }=\sqrt{\bar{\tau }_x^2+\bar{\tau }_y^2}$ is the shear stress magnitude, and $k_x=k\cos {\theta }$ and $k_y=k\sin {\theta }$, where $k=\sqrt{k^2_x+k^2_y}$ is the wavenumber magnitude. The expression is further simplified to

21 $$\begin{aligned} \bar{u}_x = \frac{\bar{\tau }\cos \theta }{2 \mu \alpha k} \end{aligned}$$

The longitudinal strain in Fourier domain, $\bar{\epsilon }_x=i k_x\bar{u}_x$, is

22 $$\begin{aligned} \bar{\epsilon }_{xx} = \frac{i \bar{\tau } \cos ^2 \theta }{2\mu \alpha } \end{aligned}$$

As an upper bound for this effect, we consider the strain amplitude induced by a tsunami propagating parallel to the cable ($\theta =0$):

23 $$\begin{aligned} \epsilon _{xx} = \frac{\tau _w}{2\mu \alpha } \end{aligned}$$

The dashed lines in Fig. 3 show the strain levels due to the applied shear stress, estimated by equation 23, as a function of frequency. For the range of frequencies relevant for tsunami detection, they remain approximately four orders of magnitude below the expected signal due to pressure. Hence, the longitudinal strain due to the shear strain at the seafloor induced by the horizontal propagation of tsunamis is expected to have a negligible contribution towards tsunami detection.

### Strain Imparted Directly on the Cable

The seafloor shear stress $\tau _w$ can also act directly on the cable, deforming it, but here we show that this second effect can be neglected. Considering a tsunami propagating parallel to the cable, the elastic strain in the cable due to the flow-induced shear stress is:

24 $$\begin{aligned} \tau _w = 2G \, \epsilon _{xz} \sim G \frac{u_x}{r}, \end{aligned}$$

where $G$ is the shear modulus of the cable and $r$ its radius. The associated longitudinal strain of the cable is

25 $$\begin{aligned} \epsilon _{xx} = \frac{\partial u_x}{\partial x} \sim \frac{r}{G} \frac{\partial \tau _w}{\partial x}. \end{aligned}$$

For a tsunami wave with wavelength $\lambda _{tsu}$,

26 $$\begin{aligned} \epsilon _{xx} \sim \frac{r}{G} \cdot \frac{2\pi }{\lambda _{tsu}} \tau _w. \end{aligned}$$

As the term $2\pi r/\lambda _{tsu}$ is very small and the elastic moduli of the cable and rock are not extremely different, the coefficient $\frac{2\pi r}{\lambda _{tsu} G}$ is orders of magnitude smaller than the coefficient $1/(2\mu \alpha )$ in Eq. 23. Thus, this additional effect can be neglected.

## Strain from a Propagating Tsunami Wave

To study the strain signal as a tsunami wave propagates, we consider a tsunami wave traveling from its source towards the coast, up to a water depth of $\textit{h}$ = 100 m. In the source area, the wave height is $\Delta h_0$ and the water depth is $h_0$. To estimate the evolution of strain during the shoaling process, we approximate the spatial variation of wave height $\Delta \textit{h}$ using Green’s law:

27 $$\begin{aligned} \Delta h = \Delta h_0 {\left( \frac{\textit{h}_0}{\textit{h}}\right) }^{1/4} \end{aligned}$$

For each water depth value *h*, the corresponding wave height $\Delta h$ is calculated. These values are then used to calculate the wavenumber *k* through equation (9), for a range of frequencies between 0.5 mHz up to 11 mHz. This is then used to compute the total strain resulting from the three effects considered so far.

**Figure 4:**
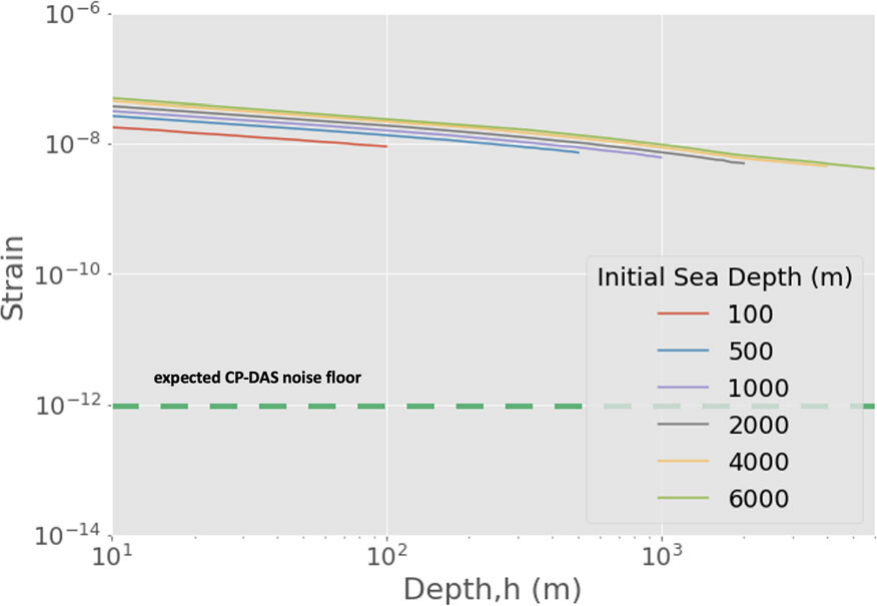
Calculated strain amplitudes from traveling tsunami waves generated at various sea depths *h* (indicated on the legend) and propagating through decreasing water depths towards the coast, to a depth of 100 m. Results are for an initial tsunami height - sea surface height anomalies (SSHA) of 0.01 m. These results are the average from 0.5 mHz to 11 mHz, and represent the strain from hydrostatic seafloor pressure. The contribution from the bottom shear stress due to the horizontal flow motion beneath a tsunami wave remain negligible at the shallow end (100 m). The green dashed line represents the noise floor of the enhanced CP-DAS unit

Figure 4 illustrates the case for an initial tsunami height - sea surface height anomalies (SSHA) of 0.01 m. Once again, we observe that the strain from hydrostatic pressure is the dominant signal along the tsunami trajectory. The contribution from shear strain becomes appreciable for large initial SSHA ($\sim 1$ meter), and only when the tsunami wave reaches shallower depths ($< 100$ meters), hence it is not expected to be a contributor for tsunami early-warning applications. The tsunami strain signal yields an SNR of approximately 200, reaffirming the feasibility of tsunami wave detection with DAS instrumentation. Towards implementation, in the following sections, we consider signals that may overlap with tsunami signals, potentially affecting detection capabilities.

## Influence of Temperature Perturbations

Temperature and strain signals co-exist in DAS data. Both phenomena affect the optical path along a fiber: strain perturbations change the fiber length and temperature fluctuations change the refraction index mainly through the thermo-optical effect. Temperature perturbations $\Delta T$ produce a proportional apparent DAS strain perturbation $\Delta \epsilon$. The proportionality factor is a function of the refractive index, thermo-optic and elasto-optic responses of the fiber silica (Fernandez-Ruiz et al., 2022; Haavik, 2022; Hartog et al., 1979). A representative estimate is:

28 $$\begin{aligned} \Delta \epsilon \approx 10^{-5} \times \Delta T \end{aligned}$$

The mean noise floor $\epsilon \approx 9.5 \times 10^{-13}$ of the DAS instrument under consideration corresponds to a temperature perturbation of approximately 95 nK. Given such fine sensitivity, it is necessary to evaluate whether thermal effects may overlap with the mechanical strain signals induced by tsunami waves. Here we consider thermal effects due to (1) background temperature fluctuations and (2) tsunami-induced fluid advection.

### Background Temperature Fluctuations

Long-term oceanographic observations in deep water indicate that temperature fluctuations at the ocean bottoms generally have dominant periods ranging from hours to weeks. Their frequency content is highly dependent on site characteristics (depth, geographic location, bathymetry, etc). For our purposes, such long-period signals can be filtered out if they do not overlap with the tsunami period range.

Frequency content and water depth can serve as a basis to distinguish strain from temperature signals in DAS seafloor data. At shallow water depths ($< 200$ m) and at short periods ($10^{-2}$ - $10^2$ s), mechanical strains from seismic, acoustic or surface gravity waves dominate over thermal fluctuations driven by the oceanic environment. This differentiation has allowed seismic phase detection with ocean-bottom DAS (e.g. Lior et al. 2020; Ugalde et al. 2022) and observations of coastal currents (Lindsey et al., 2019; Williams et al., 2019). In deep water or at long periods, thermal and mechanical DAS signals may have comparable amplitudes.

Recent studies suggest ocean-bottom DAS can record temperature. (Ide et al., 2021) interpreted DAS signals at tidal periods and with apparent propagation speeds of 0.5 m/s, recorded along a cable offshore Japan, as temperature fluctuations of a few Kelvins due to water advection driven by tides. (Williams et al., 2023) observed, along a cable off the island of Gran Canaria, the propagation of thermal fronts associated with nonlinear internal tides, with apparent velocities in the 0.1–0.5 m/s range.

In general, thermal anomalies driven by oceanographic processes are larger in shallow waters. Ocean-bottom DAS data confirms that temperature fluctuations are weaker with increasing depth. Off the coast of Toulon, France, (Pelaez Quiñones et al., 2023) found temperature fluctuations in the 0.1–1 mHz band reaching 0.1 K at depths shallower than 100 m and in the range of 0.01 to 0.001 K at further depths down to 2000 m, with apparent velocities close to 0.1 and 0.01 m/s, respectively. Off Gran Canaria, (Williams et al., 2023) observed perturbations up to about 2 K at 1.4 km depth and 0.2 K at 2.5 km depth.

The thermal DAS signals due to internal waves and tides may overlap with the mechanical DAS signals due to tsunami waves in the mHz range. From (28), fluctuations larger than 0.01 K will correspond to strain signals above $10^{-7}$, which may exceed tsunami signal amplitudes (Fig. 3). Yet, these temperature anomalies propagate about 3 orders of magnitude slower than tsunami waves, thus it should be possible to filter them out based on their apparent speed $C_x = C/\cos (\theta )$, where *C* is the phase velocity and $\theta$ is the angle between the direction of propagation of the temperature front and the cable orientation. Although oblique incidence tends to increase the apparent velocity, only a very narrow range of incidence angles, within $0.2^{\circ }$ from normal incidence, would make the temperature signal velocity comparable to tsunami velocities. Hence, frequency-wavenumber filtering based on signal speed is expected to be a viable approach.

If the cable is not straight, the wavenumber decomposition cannot be obtained by Fourier transform, and alternate strategies need to be devised. This may consist of in situ calibration of the instrument by characterizing temperature variations that may be pervasive and endemic to a given offshore location; to consider cable construction and burial, and to make key observations of the environment. Temperature fluctuations on seafloor DAS recordings may also be mitigated by burying the cable. Indeed, (Pelaez Quiñones et al., 2023) and (Williams et al., 2023) reported a lack of temperature signals along cable sections that are buried. While buried sections along telecom cables are rare, they could be a design consideration for fiber optic cables tailored for geophysical monitoring.

### Tsunami-Induced Temperature Fluctuations

In principle, seafloor temperature perturbations can arise from advection of the thermally stratified water column by a tsunami wave. However, such signals have not been observed yet. Temperature data during tsunamis has been fortuitously recorded in association with seafloor pressure observations, as part of the system that compensates for the thermal drift of pressure transducers (Eble et al., 1989; Joseph, 2011). One ocean bottom station in the 2011 $\hbox {M}_w$=9.0 Tohoku earthquake source area, at a sea depth of 1.1 km, recorded a water temperature increase of $0.19^\circ$C about 3 h after the earthquake, lasting for several hours. This temperature transient was attributed to warm water discharges, as well as a tsunami-generated turbidity current (Arai et al., 2013; Inazu et al., 2023).

During the 2003 $\hbox {M}_w$=8.3 Tokachi-oki earthquake and tsunami, a CTD (Conductivity-Temperature-Depth) station located in the source area recorded a temperature perturbation at least two hours after the mainshock, which was attributed to a benthic storm (Mikada et al., 2006). Furthermore, the temperature data at two seafloor pressure stations found no significant change in the tsunami pressure signal after applying a temperature correction (Inazu & Hino, 2011). As the temperature measurements from quartz crystal transducers are primarily intended to compensate for the thermal drift of the pressure gauge, their temperature resolution is limited. In the absence of well-resolved observations of tsunami-induced seafloor temperature changes, we turn next to back-of-the-envelope theoretical analysis.

An order-of-magnitude estimate shows that temperature changes due to deep water advection by a tsunami wave could be recorded by seafloor DAS, but would be much smaller than the strain mechanically induced by the tsunami. Considering a SSHA of 10 cm, the horizontal water particle displacement at the sea bottom is of at least 1 m (Ward, 2003). Assuming this same horizontal advection follows along a typical slope of 5% between a continental shelf and a subduction trench, it displaces water vertically by 0.05 m along the slope. Considering a generic vertical temperature gradient for the open ocean of 0.002 K/m at depths exceeding 1 km (Talley, 2011), the vertical water advection carries a temperature change of $\sim$ 0.1 mK. This value corresponds to a DAS strain $\sim 10^{-9}$, which is above the enhanced CP-DAS noise floor of $3 \times 10^{-10}$ at 1 mHz, but about two orders of magnitude below the expected strain mechanically induced by tsunami waves (Fig. 3).

## Extracting the Tsunami Signal in the Near-Field of a Large Subduction Earthquake

With the improvements to DAS instrument sensitivity at low frequencies illustrated in Fig. 1, our analysis of the expected seafloor strain due to tsunami waves (Figs. 3, 4) points to the feasibility of tsunami detection with seafloor DAS. To further demonstrate the potential contribution of DAS to TEWS, we analyze here a synthetic data set from a fully physics-based 3-D simulation of a magnitude $M_W$ 8.5 earthquake dynamic rupture and tsunami generation. This approach enables us to assess the feasibility of extracting tsunami signatures, even in the near-field of the earthquake rupture.

### Coupled Ocean-Solid Earth Simulation Setup

**Figure 5:**
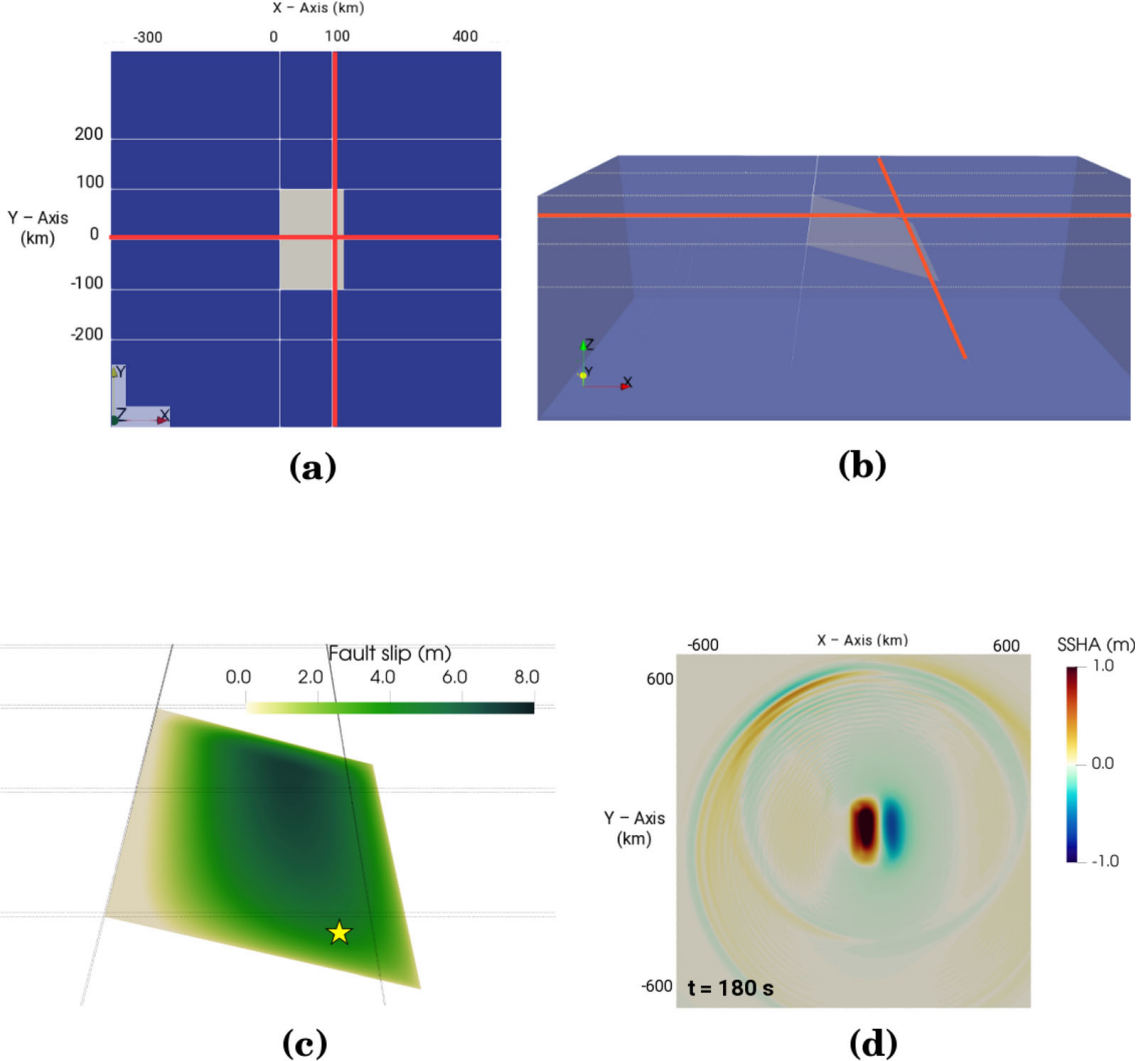
Benchmark 3-D fully-coupled simulation scenario showing location and dimensions of the planar, $16^{\circ }$-dipping fault, resembling a subducting plate, relative to the receiver matrix. The entire simulation space encompasses an area of 2750 km $\times$ 2750 km, with a uniform 2 km water layer atop the elastic medium. For clarity, **(a)** and **(b)** only show the area of 750 km $\times$ 750 km containing the fault and the surrounding receiver matrix. Receivers along each linear array are arranged in pairs separated by 50 m, respectively, which emulates commonly used DAS gauge lengths; the next adjacent receiver pair is placed 450 m apart. The simulation consists of two identical matrices, one buried 10 cm below the seafloor, whilst the other matrix is located 10 cm below the sea surface. Given the orientation of the fault, increasing positive values move towards the coast along the X-axis, whilst negative values go seaward. X and Y coordinate values are given in kilometers. The highlighted arrays at Y = 0 km and X = 100 km are used in this analysis. The earthquake nucleation patch is located at the southeast corner of the fault at 26.25 km depth as shown in **(c)**, along with the accumulated fault slip. The higher strength near the top of the fault smoothly stops the dynamic rupture as it approaches the surface. **(d)** shows the vertical water column displacements (tsunami generation) at 180 s simulation time after the onset of nucleation for the area 1300 km $\times$ 1300 km

We consider an idealized megathrust earthquake on a shallowly dipping, planar thrust fault, with non-linearly coupled tsunami generation and propagation across a compressible ocean layer with constant depth. The model setting is based on a benchmark scenario for linking dynamic rupture earthquake and tsunami simulations established in Madden et al. (2021) and Krenz et al. (2021). The fault strikes North, is 200 km wide along the strike, extends from the surface to 35 km depth, and dips eastward with a $16^{\circ }$ dip angle (Fig. 5). The ocean has a water depth of 2 km, acoustic wave speed of $\hbox {c}_{{p}}$ = 1500 m/s and density $\rho$ = 1000 kg/$\hbox {m}^3$. The solid half-space has homogeneous elastic properties representative of oceanic crust: P-wave speed $c_p = 7639.9$ m/s, shear wave speed $c_s = 4229.4$ m/s and density $\rho = 3775$ kg/$\hbox {m}^3$. Because this setup ignores the presence of shallow compliant layers, the resulting seafloor compliance signal should be understood as a lower bound.

The simulation employs a fully coupled 3-D earth and ocean model of earthquake dynamic rupture, seismic and acoustic wave propagation, whilst simultaneously solving for the tsunami (gravity) wave propagation, implemented in the simulation package SeisSol (www.seissol.org). The tsunami is modeled through linearized equations, derived by combining mass balance with a linearized equation of state and momentum balance, with gravity acting as a restoring force and an initial small perturbation about the hydrostatic rest state of the ocean (Lotto & Dunham, 2015). The simulation self-consistently computes the full wavefield in 3-D, which comprises seismic, acoustic, and surface gravity waves in elastic (earth) and acoustic (ocean) media and has been applied to the 2018 Sulawesi earthquake and tsunami in Palu Bay (Krenz et al., 2021) as well as to scenario-based modeling of a strike-slip earthquake-tsunami in North Iceland (Kutschera et al., 2024).

SeisSol is based on the arbitrary high-order derivative Discontinuous Galerkin (ADER-DG) method and optimized for modern high-performance computing infrastructure (e.g., Heinecke et al. 2014; Uphoff et al. 2017). Here, we use a fifth-order accurate scheme and an unstructured tetrahedral mesh with a minimum element size of 66 m (on the fault), consisting of $\sim$88.2 million elements. This high resolution is required to accurately resolve the process zone, the region behind the rupture front where the fault strength drops from its static to dynamic level. We simulate 10 min of combined earthquake dynamic rupture, tsunami generation, and tsunami propagation on the supercomputer SuperMUC-NG, which requires a total of $\sim$430,000 CPUh using 512 nodes (24,576 cores) for $\sim$17.5 h to resolve at least up to 6 Hz of the seismic, 2 Hz of the acoustic, and 0.2 Hz of the tsunami wavefield (Käser et al., 2008), assuming an envelope misfit accuracy of at least 90 per cent and velocities of 4229.4 m/s, 1500 m/s, and 140 m/s, respectively.

**Figure 6:**
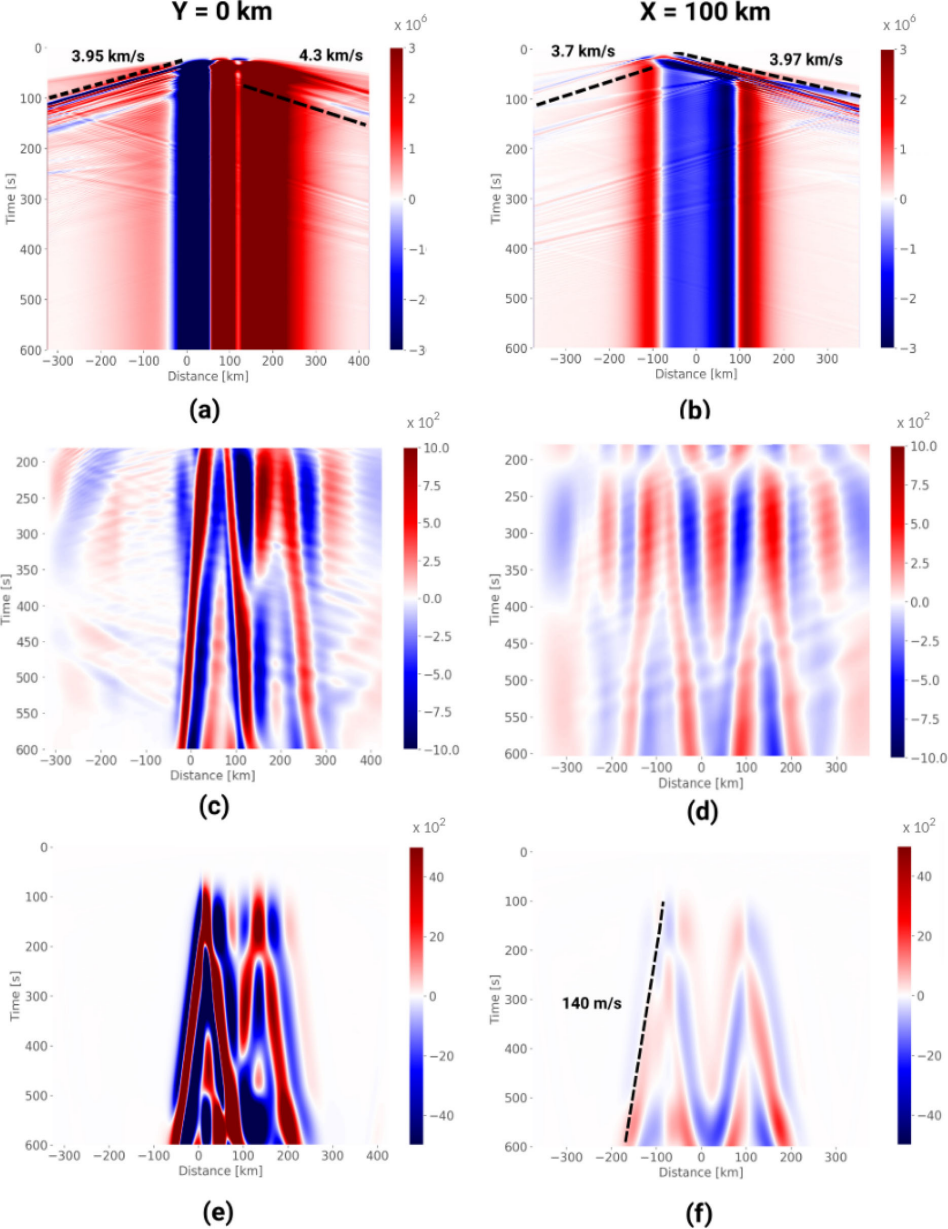
Strain signal corresponding to a trench-orthogonal array located at $y = 0$ km (left column) and a trench-parallel array at $x= 100$ km (right column). Top (a, b): Broadband strain recorded on seafloor-coupled receivers. Dashed black lines indicate seismic and surface wave velocities. Middle (c, d): same after removing the initial 180 secs, subtracting the mean of the final 180 secs (static seafloor deformation) from each receiver and filtering in the temporal and spatial domains, the seismic surface waves by bandpass filtering between 3–10 mHz onto the range of wavelengths (11–66 km) corresponding to propagating velocities between 110–200 m/s. Bottom (e, f): Estimated strain signal due to seafloor pressure (seafloor compliance and the cable’s Poisson effect), derived by subtracting the vertical seafloor displacement from the sea surface height anomalies (SSHA, recorded by receivers near the top of the water column), temporal-spatial filtered as in (c, d). Color scale represents strain expressed as a signal-to-noise ratio (SNR) relative to the noise amplitude of the DAS instrument described in section 2. The dashed black line in (d) indicates the tsunami phase velocity

The earthquake source evolves spontaneously during the earthquake dynamic rupture simulation governed by linear slip-weakening friction (e.g., Ida 1972; Harris et al. 2018). The earthquake hypocenter is located in the southeast corner of the fault at 26.25 km depth. While the fault does reach the seafloor, the rupture itself is buried; higher fault strength at shallow depth smoothly stops the rupture as it approaches the surface (Fig. 5c), with the rupture front propagating at 3.5 km/s on average.

Simulation outputs are recorded on 42,000 receivers in total, thereof 21,000 are buried 10 cm below the seafloor. They are arranged by pairs, with an intra-pair separation of 50 m, comparable to a commonly used DAS gauge length, and an inter-pair separation of 500 m. The receivers are arranged in 7 linear arrays, either parallel or orthogonal to the trench (Fig. 5a, b). The trench-parallel and trench-normal linear arrays, across and in the vicinity of the earthquake source area, represent end-member configurations of a seafloor cable. Based on our previous analysis, we focus on longitudinal seafloor strain induced by the tsunami wave only from hydrostatic pressure, namely the compliance and Poisson effects (Eqs. 7 and 8, respectively). We calculate the seafloor strain taking the finite difference between pairs of seafloor receivers as $\epsilon =\frac{u_1-u_2}{d}$, where $u_i$ is the displacement component parallel to the direction of the linear array at receiver *i*, and $d = 50$ m is the intra-pair receiver distance. The displacement is obtained by time integration of the velocity data.

To record SSHA, an additional set of receivers (another 21,000 receivers) were placed near the top of the water column, directly above the seafloor receivers. We extract the SSHA from the vertical displacement of these receivers. Seafloor pressure is calculated from Eq. 11 based on the effective water column height, obtained by subtracting vertical seafloor displacement from SSHA. We then compute the estimated strain signal due to seafloor compliance and the cable’s Poisson effect by using Eqs. 7 and 8.

### Enhancing the Tsunami Signal

The resulting seafloor strain fields for two profiles, one perpendicular to the fault (the array at $y=0$ km) and the other along the buried tip of the fault ($x=100$ km), are shown in Fig. 6 (a) and (b), respectively. The strain field is largely dominated by seismic and acoustic waves while the rupture propagates across the fault for $\sim 60$ s. The strain field continues to change after this time until it reaches final values at $t \sim 80$ s (Madden et al., 2021). From then on, the seafloor strain is dominated by the static deformation above and around the rupture area. Static displacements reach $\sim$ 3 m along the $y = 0$ km line and 30 cm along the $x = 100$ km line.

The spatial extent of the simulation comprised a large area of 2750 $\times$ 2750 km to mitigate spurious reflections originating from the imperfect absorbing boundaries of the computational domain. The amplitudes of such artificially reflected waves are small (on the order of 1e-5 to 1e-7 m). However, they induce a signal of comparable amplitude to that of the predicted tsunami-induced strain signals. These artifacts could be reduced by employing more advanced boundary conditions, which is a non-trivial problem (e.g., Duru et al. 2019). Figure 5a and b only show a subsection of the simulated area which contains the simulated receiver arrays located in the vicinity of the fault and avoids spurious reflections.

We apply the following data processing steps to visualize the tsunami signal in the simulation results. The simulated tsunami has a dominant wavelength of $\approx$ 57 km, which is much larger than the water depth. Thus, the shallow-water tsunami regime holds and we should be looking for tsunami signals propagating at speed $\textit{v} \approx \sqrt{g h}$ = 140 m/s. Within the initial 10 min simulated here, as the tsunami only propagates within the vicinity of the tsunamigenic region, detection requires techniques to discern the tsunami signals which can be orders of magnitude below the seismo-acoustic wave and static deformation signals. As exemplified in the raw broadband seafloor strain (Fig. 6a, b), the tsunami signal is not readily visible, but becomes observable after the initial 180 s using appropriate post-processing (Fig. 6c, d). We first subtract an estimate of the static strain from each channel, calculated as the mean over the final 180 s. We then apply a 40% window taper along the remainder of the time series and along each receiver array. We finally band-pass filter in the temporal domain in the frequency range of 2–8 mHz and in the spatial domain in the wavelength range of 11–66 km. The wavelength range is derived from the frequency range by dividing by a range of wave velocities around the expected tsunami speed, namely 110–200 m/s. For the $y = 0$ km array, the filtering was implemented via second order Butterworth filters. It was also necessary to remove a single receiver in the vicinity of the trench which showed a spatially abrupt change in displacement polarity. To reduce the presence of boundary reflections, cascaded second-order filters were employed for the $x = 100$ km array. Upon post-processing, we readily observe the tsunami wavefronts on the trench-parallel array (Fig. 6c) and on the trench-orthogonal array (Fig. 6d). In spite of the seismo-acoustic and static signals that dominate the broadband seafloor strains (Fig. 6a, b)—about three orders of magnitude larger than the tsunami strain signals (Fig. 6e, f)—, some processing steps allow to effectively extract the tsunami signal.

**Figure 7:**
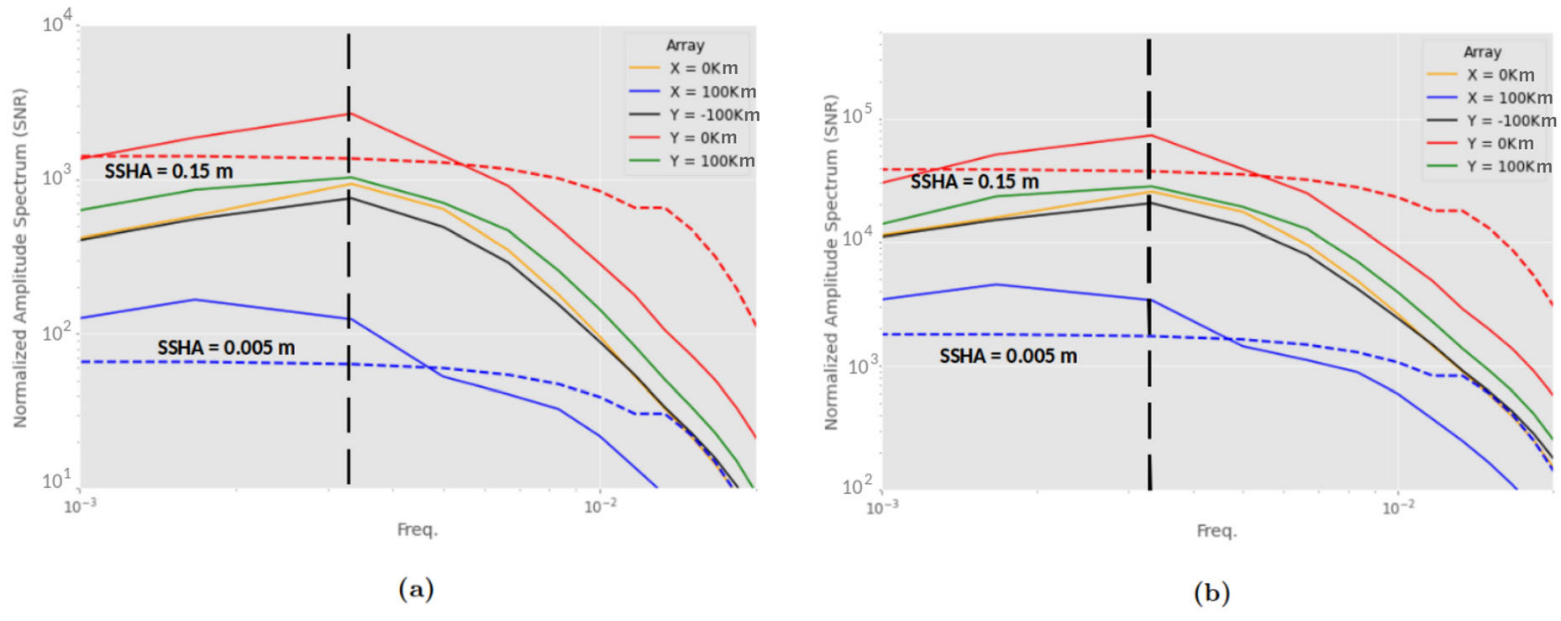
Amplitude Spectral Density (ASD) of the synthetic tsunami signal from the simulation due to (a) seafloor compliance and (b) the full hydrostatic pressure (Poisson and seafloor compliance). Pressure is derived from the effective vertical displacement, by subtracting the vertical displacement at the seafloor from the SSHA (receivers just below the sea surface). The PSD is calculated for each receiver and then averaged along each array. Power is converted to amplitude. The signal is normalized relative to instrument noise (SNR). Red and blue dashed lines follow the color assignment on the legend, where the labeled SSHA values correspond to the maximum displacement (after filtering, as described previously) observed on the given array. Such SSHA is used in Eqs. 7 and 8 to define the predicted signal from the derived model. The dashed black line marks the dominant tsunami wave frequency

### Comparison to DAS Sensitivity and to the Theoretical Model

We compute the expected DAS SNR by taking the ratio between the filtered strain and the mean noise floor of the DAS instrument shown in Fig. 1. Because this simulation did not model a seafloor cable, the contribution from Poisson’s effect is not included in the simulation data shown in Fig. 6c, d. Both seafloor compliance and Poisson’s effect are included in Fig. 6e, f, which is derived from the water column height simulation data. Among the DAS array configurations examined here, we find that the best suited for tsunami detection is located close to the source area and perpendicular to the trench.

We compare the simulated seafloor compliance signals over all receiver arrays, to the strain predicted by the theoretical model. We calculate the power spectral density of the estimated strain due to seafloor compliance at each receiver, based on their SSHA and vertical seafloor displacement, then average along each array, and take the square root to convert power to amplitude. The results are shown in Fig. 7a. The arrays at $y = {\pm }$200 km are ignored because the tsunami does not reach them during the 10 min of simulation. The SNR reaches values higher than 1000 during the whole time window along the trench-orthogonal array (Fig. 6c and curve Y = 0K in Fig. 7a) and of order 100 along the trench-parallel array (Fig. 6-d and curve X = 100K in Fig. 7-a).

The expected tsunami signal from the full hydrostatic effect (Poisson effect and seafloor compliance) is shown in Fig. 7-b. In this instance, the SNR values are an order of magnitude higher to those reported in Fig. 7a. It is worth highlighting that the solid half-space in the simulation has homogeneous elastic properties representative of oceanic crust. As this configuration does not account for the presence of shallow, compliant layers, the simulated seafloor compliance signal (Figs. 6c and 7a) should be interpreted as a lower bound.

The strongest signal is recorded on the $y = 0$ km array, which is aligned with the prevalent propagation direction of the tsunami. Overall, the theory gives an adequate order-of-magnitude estimate of the signal across all arrays. The two dashed curves in Fig. 7 are the estimated signal for two selected values of SSHA (see labels), corresponding to the maximum SSHA for the array with the largest SSHA ($y = 0$ km) and for the array with the lowest SSHA ($x = 100$ km). The theoretical and the simulation amplitudes agree in order of magnitude. Their differences are attributable to the simplifying assumptions in the theoretical estimate: the same SSHA value at all frequencies, a single tsunami propagation direction.

## Summary

Motivated by the need to advance tsunami warning systems and the fundamental understanding of tsunami processes, we have evaluated, through theoretical analysis and numerical simulations, the potential of Distributed Acoustic Sensing on seafloor fiber optic cables for direct observation of tsunami waves.

We derived first-order estimates of the seafloor DAS signals potentially generated by tsunamis. We considered two mechanisms through which the hydrostatic pressure perturbation of tsunami waves can induce longitudinal strain on a cable: the elastic deformation of the seafloor (compliance) and the Poisson effect within the cable. We also quantified two mechanisms by which the sub-horizontal deep water flow, induced by tsunami waves, can generate DAS signals: seafloor shear and temperature advection. However, we found them to have a relatively minor potential contribution in deep waters. Seafloor shear contributes significantly only at shallow depths (< 100 m) and for relatively large initial SSHA ($> 0.1$ m).

To further evaluate the use of DAS for TEWS, we carried out a full-physics simulation representative of a large earthquake rupture in a subduction zone. This simulation allowed to assess our ability to separate the tsunami contribution from the static and dynamic earthquake components, depending on their relative distance and the orientation of the cable. The observations from the cable geometries simulated illustrate the need to strategically select the placement of the cable. We found that the preferred cable orientation is aligned with the horizontal displacement most likely to occur (i.e. perpendicular to the trench), as the tsunami energy and propagation will mirror such a pattern and because DAS records longitudinal strain. While less optimal, cables perpendicular to the prevalent horizontal displacement (i.e. parallel to the trench) are still relevant for tsunami detection. We expect the overall signal processing approach presented here to be compatible in most situations, provided its parameters are tuned according to the characteristics of the cable, its orientation, and to account for the endemic characteristics of the monitored region to discern the tsunami signal, as illustrated in Fig. 6c, d.

### Current Constraints of DAS Technology for Tsunami Detection

Distributed Acoustic Sensing (DAS) presents a promising tool for direct tsunami observation via seafloor fiber optic cables. However, current implementations exhibit strain noise amplitude spectral density (ASD) levels that challenge this sensitivity, primarily due to 1/*f* noise, which dominates at low frequencies.

If the seafloor compliance and the Poisson effect on the cable represent the primary mechanisms through which DAS systems are anticipated to record the passage of tsunami waves; in order to achieve a sensitivity similar to that of a DART station (1 mm amplitude at 4 km water depth) (Mungov et al., 2013), the minimum amplitude of the strain signal to be resolved is $\epsilon \approx 5 \times 10^{-10}$ at frequencies in the order of tens of mHz. The attained values of single-channel strain noise ASD for the DAS equipment considered herein, operating at kHz sampling frequencies suggest that these water column changes can be resolved and likely enhanced using the information from the full array. Such enhancements in the low-frequency regime would directly address the limitations due to higher noise floors described by Tonegawa (2024). Furthermore, beamforming methods allowing significant processing gains, have shown to reach the threshold of tsunami signal detection (Xiao et al., 2024), despite single-channel limitations. The strain noise ASD of conventional DAS systems in this frequency range is dominated by 1/*f* noise, more specifically to the DAS unit considered herein, this is caused by reference updates and laser frequency drifts, where the latter is a common issue for all DAS instrumentation. Methods for improving the sensitivity of DAS instrumentation at these frequencies via stabilization of the frequency content of the laser pulse, have demonstrated a way to strongly mitigate instrumental noise and reach the low-frequency performance necessary for tsunami detection at the single-channel level. Towards the complete cancellation of the 1/f noise components associated with noise integration with each reference update, a novel technique has been suggested which avoids resorting to update the reference measurement, by acquiring a database comprised of a set of reference measurements along unperturbed fiber, each at a different frequency of the interrogation laser, allowing “calibrated” measurements in CP-DAS (Vidal-Moreno et al., 2022).

To optimize early warning times, it is best to detect the tsunami in the generating region however, the dynamic range of the instrument can be a concern. Seismo-acoustic signals, particularly during large earthquakes, can exceed the strain limits of the instrument ($\Delta \epsilon _{max} \approx \pm 0.4 \times 10^{-6}$), leading to temporary saturation and delayed detection. Furthermore, as exemplified in the featured simulation, cables directly over the subducting slab will also record the quasi-static seafloor displacement, these being orders of magnitude larger than the tsunami wavefield, with both having similar spatio-temporal characteristics. We show that it is still possible to retrieve the tsunami signal after appropriate filtering and after a few minutes delay of tsunami detection (3 min in our simulation), but this delay remains compatible with the design of local TEWS as tsunami waves usually take more than 10–15 min to reach the nearby coasts. The method employed here of first subtracting a static-strain estimate based on a finite averaging window (180 s) effectively introduces a high-pass filter which may partially remove or distort low-frequency components of the tsunami signal. The trade-off between delayed detection and signal fidelity should therefore be considered. Possible strategies to reduce this delay may consist of customized signal processing, monitoring for the tsunami signal from strategically selected segments of the array, away from the strongest seismic signals, or by selecting a different array placement altogether. This underscores the importance of tuning array placement and processing strategies to mitigate early saturation.

### In-situ Validation

Our simulations reveal that environmental and local site characteristics heavily influence DAS signal quality and interpretation. In our simulations, the use of a rigid, homogeneous crust provides a conservative estimate of signal strength. In reality, the presence of more compliant near-surface layers-such as sediments-will amplify strain responses to tsunami waves, particularly at low velocities. Mechanical coupling between the fiber optic cable and these shallow structural layers on the seabed is critical.

Moreover, the orientation and layout of the cable relative to likely tsunami propagation directions and seismic displacement patterns significantly affect signal strength and separation of tsunami-related strain from other sources. Cables aligned with the direction of seafloor motion (e.g., perpendicular to the trench in subduction zones) yield stronger signals. Even less optimal geometries can still contribute to detection when coupled with tailored signal processing approaches. These observations underscore the need for location-specific evaluation of DAS installations for operational tsunami early warning systems.

The burial of fiber optic cables introduces an additional design trade-off. Seafloor compliance is the dominant mechanism coupling water pressure perturbations to longitudinal strain, and improved coupling-such as that achieved by burial-can strengthen this response. A buried cable benefits from reduced motion due to ocean currents and exhibits greater stability against temperature fluctuations. However, burial also suppresses the Poisson effect, since the direct deformation of the cable by hydrostatic pressure variations is reduced. Therefore, while burial improves strain signal fidelity for compliance-driven detection, it may reduce sensitivity to the Poisson-induced component of the tsunami signal. The choice of burial depth must therefore balance environmental stability and the range of detectable strain mechanisms.

Oceanographic conditions also contribute to the background noise. DAS acquisitions blend strain, temperature, and pressure effects, necessitating robust calibration strategies. For example, low-frequency temperature perturbations can be filtered out based on their slow propagation speed, if characterized a priori; possibly using physical models or ancillary sensors for cross-validation.

In-situ validation, therefore, must include calibration procedures that account for the endemic characteristics of the region, including bathymetry, sediment type, water column structure, and local seismicity. Tailoring DAS system deployment and signal processing to these conditions is essential for operational reliability in tsunami early warning contexts.

## Conclusion

The theoretical and numerical considerations presented in this work demonstrate that there is a realistic path towards the detection of tsunami waves with DAS systems. Recent advancements in instrumental stability have shown potential in overcoming sensitivity constraints primarily due to 1/*f* noise, which dominates at low frequencies. Active laser stabilization and calibration methods free of reference-update recurrences, such as CP-DAS using multi-frequency reference databases, have been shown to mitigate instrumental noise and bring single-channel sensitivity closer to the needed thresholds.

Furthermore, in-situ environmental assessment of the DAS response is not optional but necessary. Seafloor properties, coupling conditions, and ocean dynamics all influence the system’s sensitivity and noise profile. Accounting for these factors through careful calibration and cable layout design will be key to moving from theoretical capability to operational reliability.

Instrumental stability along with signal processing, and calibration will progressively extend the effective low-frequency performance required for reliable tsunami detection. Given the widespread availability of undersea telecom cables and the advantages of DAS, i.e., low maintenance, dense coverage and sampling, real-time data; this technology stands poised to significantly augment existing tsunami monitoring systems and deepen our understanding of tsunami dynamics in the years to come.

## Data Availability

The open-source software SeisSol ( Gabriel et al. (2025); commit c2220f24;) has been used for fully coupled earthquake-tsunami modeling. The SeisSol input files for the simulation which generated the synthetic dataset described in section 7 and accompanying Python routine to process and generate Fig. 6; as well as Python routine to simulate the strain model outlined in sections 3 through 5 and generate Figs. 3 and 4 is accessible via (Becerril, 2024) hosted by Recherche Data Gouv.
